# Optimal Service Provisioning for the Scalable Fog/Edge Computing Environment

**DOI:** 10.3390/s21041506

**Published:** 2021-02-22

**Authors:** Jonghwa Choi, Sanghyun Ahn

**Affiliations:** Department of Computer Science and Engineering, University of Seoul, Seoul 02504, Korea; David13@uos.ac.kr

**Keywords:** fog/edge computing, service provisioning, service placement, service offloading, Internet of Things (IoT)

## Abstract

In recent years, we observed the proliferation of cloud data centers (CDCs) and the Internet of Things (IoT). Cloud computing based on CDCs has the drawback of unpredictable response times due to variant delays between service requestors (IoT devices and end devices) and CDCs. This deficiency of cloud computing is especially problematic in providing IoT services with strict timing requirements and as a result, gives birth to fog/edge computing (FEC) whose responsiveness is achieved by placing service images near service requestors. In FEC, the computing nodes located close to service requestors are called fog/edge nodes (FENs). In addition, for an FEN to execute a specific service, it has to be provisioned with the corresponding service image. Most of the previous work on the service provisioning in the FEC environment deals with determining an appropriate FEN satisfying the requirements like delay, CPU and storage from the perspective of one or more service requests. In this paper, we determined how to optimally place service images in consideration of the pre-obtained service demands which may be collected during the prior time interval. The proposed FEC environment is scalable in the sense that the resources of FENs are effectively utilized thanks to the optimal provisioning of services on FENs. We propose two approaches to provision service images on FENs. In order to validate the performance of the proposed mechanisms, intensive simulations were carried out for various service demand scenarios.

## 1. Introduction

The Internet of Things (IoT) is a new wave significantly affecting our lives in various areas including smart energy grids, smart factories, smart cities, and smart farms [1]. IoT is accomplished by sensing (monitoring) the target environment, collecting and analyzing the sensory information and actuating based on the feedback information from the analysis. The data produced in the IoT environment may be enormous and require intensive computation such as for deep learning-based prediction. Hence, the IoT data tend to be transmitted to the remote cloud data center (CDC) for storage and computation [2,3,4]. Many IoT applications have stringent timing requirements which may not be met by cloud computing because of long-distance message transmissions through the network between CDC and end devices (aka IoT devices). For the reduced latency between CDC and end devices, the concept of fog computing has been introduced by Cisco [5]. In fog/edge computing (FEC), fog/edge nodes (FENs) located in the proximity of end devices provide services to them in lieu of CDC without causing a long delay by executing the lightweight virtualized service images pre-allocated from CDC [5,6,7]. However, due to the limited capacity (CPU, storage, etc.) of FENs, only a subset of service images can be placed on each FEN [8,9].

In the previous work on service provisioning (or placement) in FEC [10,11], a service is placed on the FEN satisfying the given service requirement for the execution of the service, assuming that the corresponding service image is already installed in the FEN. Service provisioning, service placement and service offloading are interchangeably used for installing service images and executing them on FENs or CDC instead of end devices. However, due to the limited capability of a FEN, it is infeasible to place all the services on an FEN. Even in the case that FENs install service images on demand for offloaded services, the resource of FENs may not be efficiently utilized because of patchwork-like service image placement [12]. In addition, depending on the IoT applications, the type of services requested may vary. For example, in an area occupied by smart factories, the service analyzing the sensed data from a specific machine for the detection of malfunctions will be requested by the factories equipped with the machine. Placing service images on FENs in the per-service request-based manner may result in the improper resource usage of FENs due to redundantly placed service images. If we can optimally provision service images on FENs based on the pre-obtained service demands (service demands and service requests are interchangeably used in this paper), the resource utilization of FENs can be optimized. To our knowledge, this issue has not been addressed to date and thus, we formulate the problem of optimal service provisioning (SP) in the FEC environment. In our work, we adopted “service provisioning” instead of service placement in the sense that service provisioning means service placement with planning.

For the scalability of the FEC environment, FENs are located hierarchically near to end devices and in between end devices and CDC which is the last resort of providing services to end devices. The following two cases (c1) and (c2) are not desirable from the perspective of resource utilization and latency, respectively:

(c1) For a sporadically requested service, the corresponding service images are redundantly placed on multiple FENs.
(c2) For the coverage of multiple requests of a service, the corresponding service image is provisioned on a FEN at a high level of the FEN hierarchy.

If we consider both the resource usages and locations of FENs and the locations of end devices requesting services, we could avoid the above-mentioned cases. The consideration of only the case (c1) (i.e., the resource utilization of FENs) may result in the case (c2) (i.e., long delay), and vice versa. Therefore, in provisioning service images in the FEC environment, we must consider both the resource usages and locations of FENs and the delay requirements and locations of service requesters. In this paper, we propose two SP approaches by extending our previous work [12]; the first one is on the basis of the number of service requests from end devices and the second one is based on the locations of end devices requesting services. We can assert that the proposed FEC environment is scalable because more service images can be accommodated by FENs thanks to the optimal provisioning of service images on them. For the performance evaluation of the proposed mechanisms, we perform simulations for various service requesting scenarios and analyze the performance in terms of the total number of service images provisioned on FENs and the total number of service requests not accommodated by FENs.

The rest of the paper is organized as follows. In Section 2, we describe the related work on the service provisioning, offloading or placement on FENs. In Section 3, the problem of service provisioning FENs is formulated as a 0–1 integer linear programming problem for the proof of the NP-hardness of the problem and then, our two approaches are described in detail. The performance of our mechanisms is evaluated in Section 4 from the simulation results. Finally, Section 5 concludes this paper.

## 2. Related Work

Services can be offered to end devices via computation offloading in which CDC or FENs perform computation in lieu of end devices. SP on FENs is the pre-allotment of the resources, like CPU, RAM and storage, of FENs to service images for computation offloading [6,7,8]. For the sake of effective computation offloading, SP on FENs must be properly carried out beforehand.

In the FEC environment, the service provisioning, placement or service offloading has been studied by many researchers with different objectives and considerations, like power consumption [13], quality of experience (QoE) [14,15,16], migration [17], network perspective [18], service atomization [19], etc. The authors of [19] proposed strategies for offloading service executions by considering the resources of CDC and FENs. For that, they introduced the concept of service atomization and parallel resource allocation. By service atomization, complex IoT services are divided into smaller atomic services which, then, can be executed on multiple FENs sequentially or parallelly in a distributed way. In their scheme, end devices offload service executions to CDC or FENs according to network and processing demands.

In [18], the application provisioning problem in the fog-cloud environment is studied from a network perspective to guarantee the quality of service (QoS) of application (service) data streams in terms of transmission delay and bandwidth for each application. Application provisioning is to find the host node (i.e., FEN) and data routing for a specific application. The authors formulate the issue as the single application provisioning (SAP) and the multi-application provisioning (MAP) problems. SAP determines the path to the FEN satisfying the bandwidth and delay requirements of data traffics from end devices for one application. MAP is the extension of SAP for multiple applications from the perspective of network link capacity (bandwidth) usage.

In [11], the deployment of multi-component application in the fog hierarchy is defined as the components deployment problem (CDP) which determines candidate FENs to be deployed with the components of an application according to application-specific QoS requirements of end devices on latency and bandwidth, software and hardware capabilities of FENs and business policies. They target to deploy large-scale applications composed of multiple components that can be independently deployable and work together for the infrastructure and prove that the CDP problem is NP-hard by the reduction from the subgraph isomorphism problem (SIP) [20]. The CDP problem is to find the optimal deployment of a single application (service) based on the service requests of end devices in a centralized manner.

Both [18] and [11] determine the FENs to be provisioned with services in a centralized way for a given set of service requests, similar to ours. However, theirs take account of each individual FEN, not of entire FENs, from the perspective of resource usage. The consideration on the capacity of each FEN is just for checking the relevant constraint, but the consideration on the total resource usage of all the FENs is for optimizing the overall resource usage of FENs. Thus, the latter is more appropriate for enhancing the scalability of the fog infrastructure in terms of resource usage.

Related to the resource provision of FENs, the authors of [14,15] proposed a FEC architecture composed of fog colonies each consisting of one fog orchestration control node and multiple fog cells. The fog orchestration control node is a fog cell with extended functionalities like managing fog cells in the same fog colony and other connected control nodes. The fog cell is the software components running on an FEN. The fog orchestration control node keeps all the service implementations (i.e., service images) in its service registry, located in the low-cost abundant storage unit, and deploys the corresponding service image to the fog cell covering the service requester on demand. In this way, the constrained resources of FENs are effectively utilized. The fog cell for a service request is determined based on the resources of FENs and the resource and delay requirements of the service request such that the resource requirement is satisfied with the minimum delay. In the scheme, all the service images are kept in the storage of the fog orchestration control node and deployed on FENs per service request by the control node. This will incur overwhelming communication overhead and delay in deploying service images on demand from fog orchestration control nodes to fog cells, and the requirement on the fog orchestration control node to be installed with all the service images is infeasible or, at least, inefficient if plenty of services are to be deployed in the fog.

In this paper, we aim to formulate and resolve the problem of minimizing the total number of service images provisioned on FENs in order to optimize the overall resource usage of FENs.

## 3. Service Provisioning for Fog/Edge Nodes

### 3.1. System Environment and Problem Description

For the simplicity of problem formalization, we assume that there is one CDC and all the service demands are accommodated by the FENs. Each service demand is assumed to require the same amount (e.g., one capacity unit) of resources even though service demands are heterogeneous in reality. The network link capacity is assumed to be sufficient for the delivery of the data generated by the service demands (i.e., we do not consider the network link capacity as a constraint in the problem).

We define the notations needed for the problem formalization in Table 1. S is the set of the services, $\left\{s_{1},\ldots ,s_{\alpha }\right\}$, and F is the set of the FENs, $\left\{f_{1},\ldots ,f_{\beta }\right\}$, and U is the set of the end devices, $\left\{u_{1},\ldots ,u_{\gamma }\right\}$, and D is the service demand matrix, $\left[d_{ik}\right]$, where $d_{ik}$ is 1 if the service $s_{i}$ is demanded by the end device $u_{k}$.

The problem of the optimal service provisioning (SP) in the FEC environment can be defined as follows:

(1) $$\mathrm{Minimize}\;\sum_{i\in \left\{1,\ldots ,\alpha \right\}}\sum_{j\in \left\{1,\ldots ,\beta \right\}}x_{ij}$$

(2) $$\mathrm{Subject}\;\mathrm{to}:\;\sum_{i\in \left\{1,\ldots ,\alpha \right\}}x_{ij}\leq c_{j},\forall \;j\in \;\left\{1,\ldots ,\beta \right\}$$

(3) $$x_{ij}=\;\left\{\begin{array}{c} 1,\;\mathrm{if}\;a\;\mathrm{service}\;\mathrm{image}\;\mathrm{of}\;s_{i}\;\mathrm{is}\;\mathrm{provisioned}\;\mathrm{on}\;f_{j}\\ 0,\;\mathrm{otherwise} \end{array}\right.$$

(4) $$x_{ij}\in \;\left\{0,\;1\right\},\;\forall \;i\in \;\left\{1,\ldots ,\alpha \right\},\;j\in \;\left\{1,\ldots ,\beta \right\}$$

(5) $$\sum_{i\in \left\{1,\ldots ,\alpha \right\}}\sum_{k\in \left\{1,\ldots ,\gamma \right\}}d_{ik}=\sum_{i\in \left\{1,\ldots ,\alpha \right\}}\sum_{k\in \left\{1,\ldots ,\gamma \right\}}b_{ik}$$

(6) $$b_{ik}=\left\{\begin{array}{c} 1,\;\mathrm{if}\;\exists \;j\in \left\{1,\ldots ,\beta \right\}\;\mathrm{satisfying}\;0<\tau_{jk}\times x_{ij}\times d_{ik}\leq T_{i}\\ 0,\;\mathrm{otherwise} \end{array}\right.,\;\forall \;i\in \;\left\{1,\ldots ,\alpha \right\}\;\mathrm{and}\;k\in \;\left\{1,\ldots ,\gamma \right\}$$

(7) $$b_{ik}\in \;\left\{0,\;1\right\},\;\forall \;i\in \;\left\{1,\ldots ,\alpha \right\},\;k\in \;\left\{1,\ldots ,\gamma \right\}$$

Equation (1) is the objective function of minimizing the total number of the service images provisioned on the FENs. Equation (2) is for the capacity constraint, termed Condition-C, and Equations (5) and (6) are for the delay constraint, termed Condition-D. The Equations (3), (4), (6) and (7) define the binary variables $b_{ik}$ and $x_{ij}$. $x_{ij}=1$ indicates that a service image of $s_{i}$ is deployed in $f_{j}$. Equation (2) checks whether each FEN accommodates service demands within its maximum capacity $c_{j}$. Equation (5) checks for whether the delay requirements of all the service demands in D are satisfied. In Equation (6), the binary variable $b_{ik}$ is set to 1, for the service demand $d_{ik}$ with 1, if there exists at least one FEN with a service image of $s_{i}$ that satisfies the maximum delay requirement of $T_{i}$, i.e., $0<\tau_{jk}\times x_{ij}\times d_{ik}\leq T_{i}$.

Because the optimal SP problem was formulated as a 0–1 integer linear programming problem, the problem is NP-hard. Thus, we propose two heuristic mechanisms that determine how to deploy service images on FENs according to the collected service demands from end devices.

### 3.2. Logical Fog Network

**Figure 1:**
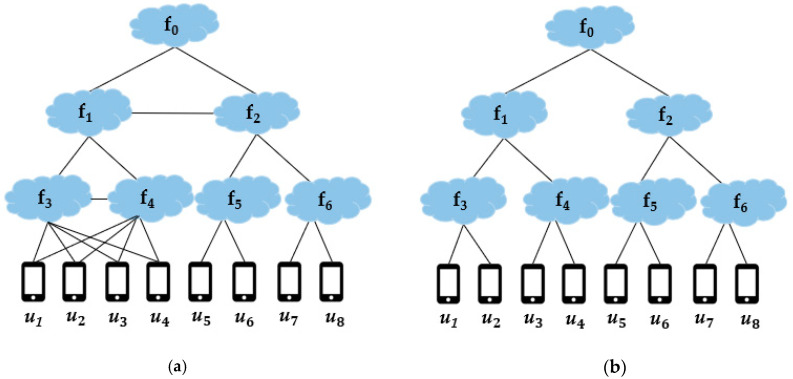
Logical fog network: (**a**) an example of a physical network; (**b**) the logical fog network of (**a**).

CDC determines the FENs to be installed with specific service images based on the service demands of the end devices. The locations of the FENs can be anywhere in the given network, determined by the deployment strategy which is out of the scope of this paper. In general, the physical network topology of the FEC environment is a mesh which increases the computational complexity of determining the optimal FENs to be provisioned with service images. Therefore, in order to simplify the problem, we form a logical tree topology of the FENs rooted at CDC, called a logical fog network [12], $N_{fog}$, in a hierarchical manner, as shown in Figure 1b for the physical network in Figure 1a in which CDC is labeled as $f_{0}$ for the sake of convenience. Because $N_{fog}$ is a subnetwork of the given physical network and the tree topology is a special case of the mesh topology, the SP problem for $N_{fog}$ is also NP-hard.

In $N_{fog}$, if an end device is in the coverage area of a FEN, we say that the end device is in the direct coverage of the FEN or the FEN directly covers the end device. If an FEN is an ancestor (including the parent) of the FEN directly covering an end device, the ancestor FEN is said to cover the end node indirectly. In $N_{fog}$, all the FENs on the path from CDC to the directly covering FEN of an end device cover the end device.

A service requester is an end device requesting the corresponding service. The data from a service requester can be handled by any FEN, with the service image and satisfying the requirements, both Condition-C and Condition-D, of the service requester on the path from the directly covering FEN up to CDC in $N_{fog}$. Each FEN, located in between the service requester and the service handling FEN, just delivers the data from the service requester to its parent FEN for the proper processing of the data.

We assume that CDC has the information on $N_{fog}$ including the end devices directly covered by the FENs and the information of the locations and the capacities of the FENs. For the provisioning of service images in $N_{fog}$, we assume that CDC has unlimited capacity such that any requirements of the end devices, except for latency, can be accommodated—that is, if there exist any service requests not accommodated by any FENs, CDC can cover those service requests.

The logical fog network, $N_{fog}$, is constructed as follows:

[Step N1] The initial $N_{fog}$ is null and the FENs are sorted in the decreasing order of the capacity.
[Step N2] CDC is first included in $N_{fog}$ as the root and the level of CDC is set to 0.
[Step N3] From the sorted list of the FENs obtained at [Step N1], the first k FENs are selected as the child nodes of CDC. Γ is the set of the child FENs of CDC. The value of the parameter k has to be properly decided by the fog network designer.
[Step N4] The delay of the link between an FEN in Γ and CDC is adjusted to a value less than its original delay (e.g., a half of the original delay).
[Step N5] Each FEN in F−Γ is added to $N_{fog}$ via the minimum delay (shortest) path to CDC.

In [Step N3], the criterion for selecting the FENs in Γ is the capacity because an FEN with more capacities can have more service images installed and a higher possibility of covering more end devices. In [Step N5], the criterion for adding the FENs in F−Γ to $N_{fog}$ is the latency because the main purpose of FEC is reducing the latency from end devices. After [Step N5], the obtained $N_{fog}$ is a logical tree rooted at CDC with the FENs in Γ directly connected to CDC and with each FEN in F−Γ connected to CDC via the shortest path. The reason for the link cost (delay) adjustment in [Step N4] is to make the possibility of the FENs in F−Γ to go through the FENs in Γ to get to CDC. From this, we can achieve the load balancing effect by making high-capacity FENs share the burden of computing load with CDC. Through the procedure of [Step N1]∼[Step N5], we can obtain a logical fog network that is appropriate for less service images provisioned thanks to the tree topology and for lower delays from end devices to FENs thanks to the shortest path branches. In the following Section 3.3, we described two mechanisms finding the right FENs, based on $N_{fog}$, that can accommodate the service images demanded by the end devices with the aim of minimizing the number of service images provisioned.

### 3.3. Service Provisioning Based on Service Demands

In this subsection, we propose two heuristic SP mechanisms, based on $N_{fog}$, in which the amount of service demands for a FEN is the major factor to be considered in placing a service image on the FEN. The first SP mechanism provisions the highest-level FEN, satisfying the conditions, Condition-C and Condition-D, of the most-demanding service, with the service so that the coverage of the FEN for the service can be maximized. Thus, the first SP mechanism is named as the maximal coverage-SP (MC-SP) mechanism. The MC-SP mechanism performs well if the distribution of the end devices demanding a specific service is uniform in the logical fog network. However, if we consider an industrial area with many smart factories requesting a specific service, the MC-SP mechanism may not perform optimally. In this situation, it is desirable to place the corresponding service image near the area. Thus, in our second mechanism, we take into consideration the locations of the end devices demanding a specific service in finding the right FEN to be placed with service images. The second mechanism is named as the flexible coverage-SP (FC-SP) mechanism. In the Section 3.3.1 and Section 3.3.2, the MC-SP and the FC-SP mechanisms are described in detail, respectively. For the simplicity of the description of the mechanisms, we assume that, in $N_{fog}$, there exists at least one FEN satisfying Condition-C and Condition-D of each service in S.

#### 3.3.1. Maximal Coverage-Service Provisioning Mechanism

Before we describe the procedure of the MC-SP mechanism, the FENs in F are rearranged to $\overset{˘}{F}=\langle f_{1}^{\prime },\ldots ,f_{\beta }^{\prime }\rangle$ such that the index of an FEN at the level q in $N_{fog}$ is smaller than that of a FEN at a level lower than q. That is, $f_{1}^{\prime }∼f_{k}^{\prime }\;\left(k=1,\ldots ,\beta \right)$ are the children FENs of CDC in $N_{fog}$ and $f_{\beta }^{\prime }$ is at the lowest level of $N_{fog}$. We define the coverage matrix $V_{fog}$ representing $N_{fog}$ such that the element of $V_{fog}$, $v_{kj}$, is set to 1 if the end device $u_{k}$ is covered by the FEN $f_{j}^{\prime }$. With given the service demand matrix D, $W=\left[w_{ij}\right]=D\times V_{fog}$ is the α×β matrix whose element $w_{ij}$ indicates the total demands on $s_{i}$ from the end devices covered by $f_{j}^{\prime }$. The notations for the SP mechanisms are listed in Table 2.

**Figure 2:**
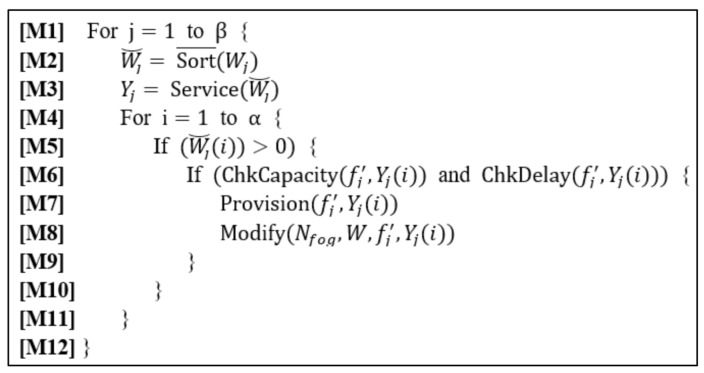
The procedure of the maximal coverage-SP (MC-SP) Mechanism.

The procedure of the MC-SP mechanism with given $N_{fog}$, $\overset{˘}{F}$ and W is described in Figure 2.

In Figure 2, the jth column of W is denoted as $W_{j}$ and the ith element of $W_{j}$ as $W_{j}\left(i\right)$. $\overset{˘}{W_{j}}$ is the sorted list of $W_{j}$, in the decreasing order of the amount of demands, which is obtained by carrying out the function $\bar{\mathrm{Sort}}\left(W_{j}\right)$ (see **[M2]**). $Y_{j}$ is the corresponding service list of $\overset{˘}{W_{j}}$ obtained from the function $\mathrm{Service}(\overset{˘}{W_{j}})$ (see **[M3]**). That is, $Y_{j}\left(i\right)$ has the information of the service whose demands imposed on $f_{j}^{\prime }$ is the ith largest among the services in $W_{j}$. The outer for-loop checks for each FEN from the highest to the lowest level of $N_{fog}$ and the inner for-loop checks for each service from the list in $Y_{j}$. That is, for $f_{j}^{\prime }$, the larger the demands on a service, the earlier the service is checked for its provisioning in $f_{j}^{\prime }$. The condition in **[M5]** is for excluding those services with no demands from being provisioned in $f_{j}^{\prime }$. In **[M6]**∼**[M7]**, the provision of the service $Y_{j}\left(i\right)$ in $f_{j}^{\prime }$ is performed if the provision satisfies the Condition-C and the Condition-D of $Y_{j}\left(i\right)$. The functions $\mathrm{ChkCapacity}(f_{j}^{\prime },\;Y_{j}\left(i\right))$ and $\mathrm{ChkDelay}(f_{j}^{\prime },\;Y_{j}\left(i\right))$ check Condition-C and Condition-D, respectively, and return the true or false value according to whether the corresponding requirement is satisfied or not. The function $\mathrm{Provision}(f_{j}^{\prime },Y_{j}\left(i\right))$ in **[M7]** provisions the FEN $f_{j}^{\prime }\;$ with the service image of the service $Y_{j}\left(i\right)$. The function $\mathrm{Modify}(N_{fog},\;W,f_{j}^{\prime },Y_{j}\left(i\right))$ in **[M8]** removes the demands on the service $Y_{j}\left(i\right)$ from the FENs which are the descendants of $f_{j}^{\prime }$ because all the service demands are resolved by $f_{j}^{\prime }$.

**Figure 3:**
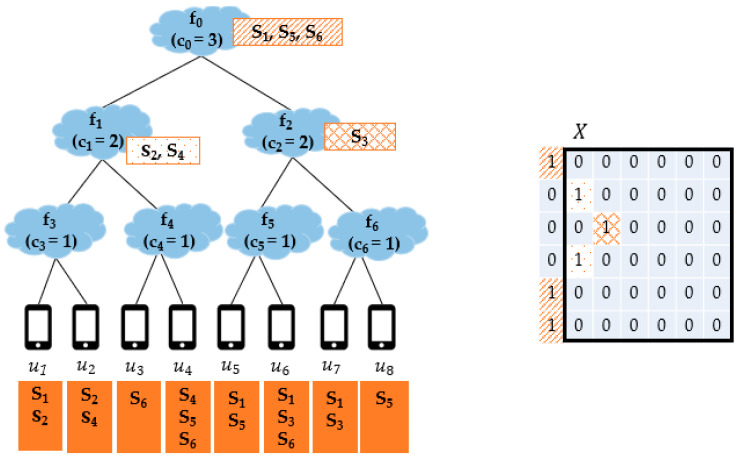
The service provisioning result of the MC-SP mechanism.

Figure 3 is a simple example showing $N_{fog}$ with the FENs $f_{1},\ldots$, $f_{6}$ and the end devices $u_{1},\ldots$, $u_{8}$ and the services $s_{1}$, …, $s_{6}$. The service demands of each end device are listed in its corresponding box and the capacity of $f_{j}$ is indicated by $c_{j}$. In this example, to focus more on SP from the aspect of Condition-C, all the FENs are assumed to satisfy Condition-D of $s_{i}$ for all $i\in \left\{1,\;\ldots ,\;6\right\}.$ For simplicity, $f_{0}$ is also assumed to satisfy the Condition-Ds of all services. In the figure, we can see the result of applying the MC-SP mechanism, and the service placement matrix X = [$x_{ij}$], indicated by a black solid line box, where $x_{ij}$ = 1 implies that $s_{i}$ is provisioned on $f_{j}$. Since all FENs satisfy Condition-D of each service, services are placed from $f_{0}$ to $f_{6}$. In MC-SP, the service with more demands has a higher chance to be placed on. In this example, $f_{0}$ can accommodate at most three services and $s_{1},\;s_{5},\;s_{6}$ have demands of 4, 3, 3, respectively, so $s_{1},\;s_{5},\;s_{6}$ are provisioned on $f_{0}$. After that, the demands on $s_{1},\;s_{5},\;s_{6}$ are removed from the other FENs. Then, services $s_{2},\;s_{4}$ are placed on $f_{1}$ and $s_{3}$ is placed on $f_{2}$.

#### 3.3.2. The Flexible Coverage-Service Provisioning Mechanism

The MC-SP mechanism takes into consideration only the hierarchical topology of $N_{fog}$ in the provisioning service images on FENs. This mechanism is the lack of the awareness of the patterns of service demands which may depend on some geographical, industrial, or social characteristics. Thus, in this subsection, we propose the FC-SP mechanism, which adaptively places service images on FENs according to the pattern of service demands. This is well suited for the situation with uneven service demands in a limited area. For this situation, an FEN near to the area is best placed to resolve the demands. Thus, we adopt the lowest-level common ancestor (LCA) FEN, in $N_{fog}$, of the end devices demanding a service as the candidate to be provisioned with the corresponding service image. If the LCA FEN *f* of a service *s* is installed with the service image of *s*, then those end devices having *f* as the LCA FEN demanding *s* can be commonly supported by *f*. In the FC-SP mechanism, the pattern of service demands is estimated by using the Shannon entropy [21]. The preference of a service image to be provisioned is determined based on the total amount of demands imposed on the FEN which is currently considered for the service provisioning. The additional notations for the FC-SP mechanism are listed in Table 3.

**Figure 4:**
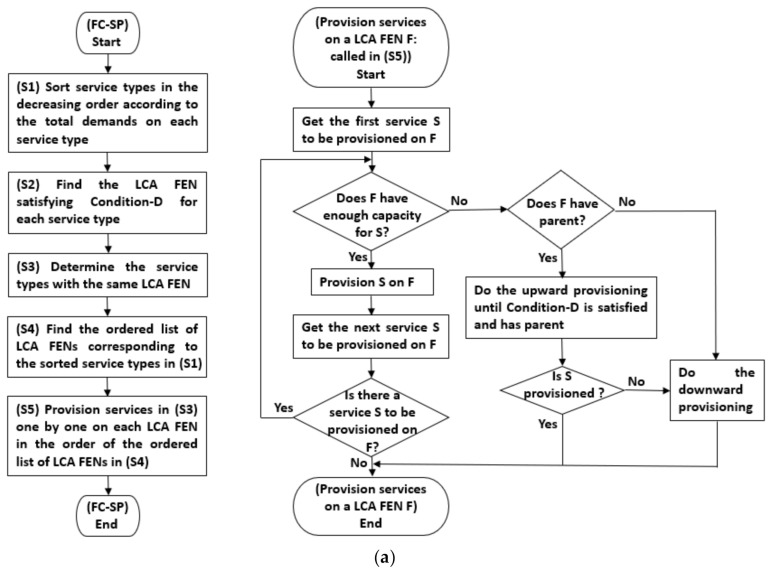

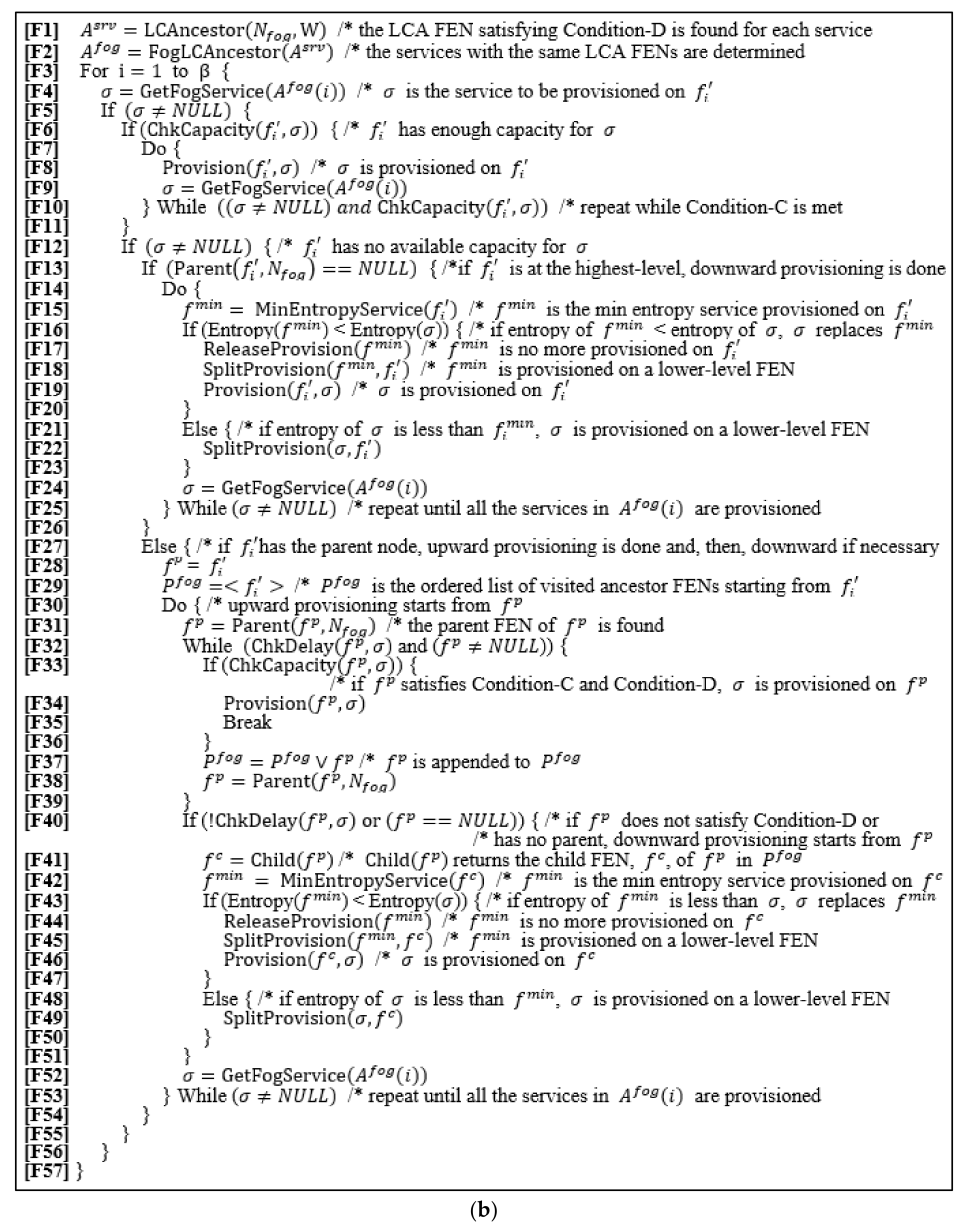
The procedure of the FC-SP mechanism: (**a**) described in flowcharts; and (**b**) described in pseudocode.

The procedure of the FC-SP mechanism with given $N_{fog}$ and W is described in detail in Figure 4. Here, we assume that, in $N_{fog}$, there exists at least one FEN satisfying Condition-C and Condition-D of each service in S. The pseudocode description in Figure 4b is somewhat lengthy and complicated because lots of notations were used, so we provided a simpler form of description, flowcharts, in Figure 4a.

The total demands on $s_{i}$ is $z_{i}=\underset{j\in \left\{1,\ldots ,\beta \right\}}{\sum }w_{ij}$, $z_{i}\in Z$. The elements in Z are sorted in the decreasing order, resulting in the sorted list $\overset{˘}{Z}$. The corresponding service list of $\overset{˘}{Z}$ is $\overset{˘}{S}=\overset{˘}{s_{1}},\ldots ,\overset{˘}{s_{\alpha }}$. For each service $\overset{˘}{s_{i}}$ in $\overset{˘}{S}$, the LCA FEN satisfying Condition-D is determined by the function $\mathrm{LCAncestor}\left(N_{fog},\;W\right)$ and the ordered list of the LCA FENs for $\overset{˘}{S}$ is $A^{srv}$. Then, the function $\mathrm{FogLCAncestor}\left(A^{srv}\right)$ returns $A^{fog}$ whose ith element, $A^{fog}\left(i\right)$, is the ordered list of the services, in the decreasing order, whose LCA FEN is $f_{i}^{\prime }$. $\mathrm{GetFogService}\left(A^{fog}\left(i\right)\right)$ in **[F9]** returns a service (the current to-be-provisioned service σ) one by one from $A^{fog}\left(i\right)$ per call. In **[F7]**∼**[F10]**, the services in $A^{fog}\left(i\right)$ are provisioned on $f_{i}^{\prime }$ while the Condition-C of $f_{i}^{\prime }$ is satisfied. If the condition in **[F12]** is met, the service σ cannot be provisioned on $f_{i}^{\prime }$ because of the capacity shortage. Then, according to the status of $f_{i}^{\prime }$, upward provisioning (see **[F31]**∼**[F39]**) and/or downward provisioning (see **[F22]**, **[F41]**∼**[F50]**) is performed. In the case that σ cannot be provisioned on $f_{i}^{\prime }$ and $f_{i}^{\prime }$ is located at the highest level of $N_{fog}$, σ may replace the already provisioned service with the minimum entropy, $f^{min}\;$ found by calling $\mathrm{EntropyService}\left(f_{i}^{\prime }\right)$, if σ‘s entropy is larger than the minimum entropy, i.e., $f^{min}$’s entropy (see **[F16]**∼**[F20]**), or be provisioned on a lower-level FEN (by calling $\mathrm{SplitProvision}\left(\sigma ,f_{i}^{\prime }\right)$, i.e., the downward provisioning of **[F22]**). Or, if σ cannot be provisioned on $f_{i}^{\prime }$ and $f_{i}^{\prime }$ is not located at the highest level of $N_{fog}$, the upward provisioning of **[F31]**∼**[F39]** is performed first and if the upward provisioning fails (that is, if all the ancestors of $f_{i}^{\prime }$ cannot provision σ), the downward provisioning of **[F41]**∼**[F50]** is performed. This process is repeated until all the service images demanded by end devices are provisioned on FENs. Here, $P^{fog}$ is the set of FENs fully provisioned with services.

In the FC-SP mechanism, the Shannon entropy of a service is used as a metric for the provisioning priority of the service. In Figure 4, the function $\mathrm{Entropy}\left(\sigma \right)$ is called when σ cannot be provisioned on $f_{i}^{\prime }$ because of Condition-C being not satisfied at $f_{i}^{\prime }$. $\mathrm{Entropy}\left(\sigma \right)$ returns the Shannon entropy of σ by using the following Equation (8) which reflects the degree of the distribution of the end devices requesting σ:

(8) $$\mathrm{Entropy}\left(\sigma \right)=-\sum_{f^{\prime }\in F^{\prime }}^{\left|F^{\prime }\right|}p_{f^{\prime }}^{\sigma }\log_{2}\left(p_{f^{\prime }}^{\sigma }\right),\;\mathrm{where}\;F^{\prime }\subseteq F$$

In Equation (8), first, $F^{\prime }$ is determined for σ, which is the set of the descendent LCA FENs of the LCA FEN currently failed in provisioning σ. $p_{f^{\prime }}^{\sigma }$ is obtained by dividing the total demands on σ at $f^{\prime }$ by the total demands on σ at the FENs in $F^{\prime }$.

**Figure 5:**
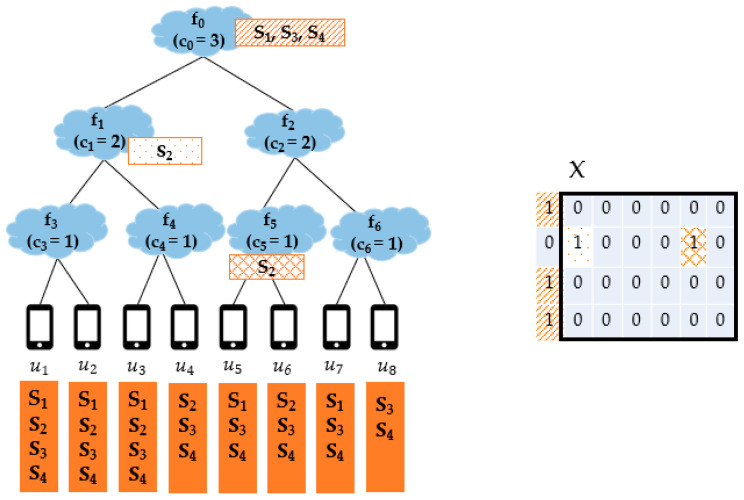
The service provisioning result of the FC-SP mechanism.

Figure 5 shows an example of applying the FC-SP mechanism to $N_{fog}$ of Figure 1b with the result of the service placement matrix X. Here, we also assume that all FENs including $f_{0}$ satisfy Condition-Ds of all services. On $f_{0}$, services $s_{3}\;\mathrm{and}\;s_{4}$ are provisioned first since each of them has demands of 8 which is the largest. After that, because $s_{1}\;\mathrm{and}\;s_{2}$ have the same amount of total demands of 5, respectively, the Shannon entropy at $f_{0}$ is calculated for $s_{1}\;\mathrm{and}\;s_{2}$. For that, $F^{\prime }$ is determined for $s_{1}\;\mathrm{and}\;s_{2}$, respectively. $F^{\prime }$ of $s_{1}$ is {$f_{1}$, $f_{2}$} and $F^{\prime }$ of $s_{2}$ is {$f_{1}$, $f_{5}$}. Then, the entropy of $s_{1}$ is “−(3/5 $\log_{2}$(3/5) + 2/5 $\log_{2}$(2/5) (about 0.9709506)” because the total demands on $s_{1}$ at $f_{1}$ is 3 and the total demands on $s_{1}$ at $f_{2}$ is 2. The entropy of $s_{2}$ is “−(4/5 $log_{2}$(4/5) + 1/5 $log_{2}$(1/5) (about 0.7219281)” because the total demands on $s_{2}$ at $f_{1}$ is 4 and the total demands on $s_{2}$ at $f_{5}$ is 1. Because $s_{1}$ has a higher entropy than $s_{2}$, $s_{1}$ is provisioned on $f_{0}$ and $s_{2}$ is provisioned on $f_{1}\;\mathrm{and}\;f_{5}$ by the downward provisioning of **[F22]**. The higher the entropy is, the more the service demands are distributed. Thus, by provisioning more distributed services on FECs at higher levels of $N_{fog}$, the capability of covering service demands can be enhanced.

## 4. Performance Evaluation

**Figure 6:**
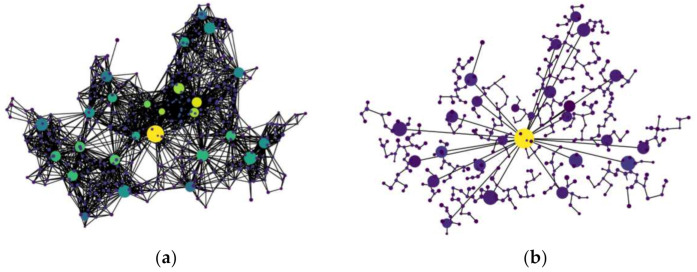
An example fog network for simulations: (**a**) a physical network with FENs in a mesh topology; and (**b**) a logical representation of the physical network of (**a**) in a tree topology [12].

Simulations were performed by using the NetworkX package [22] and Python. The simulation network environment is similar to that of [12]. The simulation network is obtained by designing an example of physical fog/edge network based on the administrative/population information obtained from Seoul open data center [23], South Korea, as shown in Figure 6a. The physical fog/edge network is built by locating one CDC (the largest dot in the figure) at the geographic center of Seoul and by locating 449 FENs according to the two-level administrative districts of Seoul. The capacity of a FEN is determined as proportional to the number of households of the corresponding administrative district. In the figure, the dots except for the largest dot (i.e., CDC) are FENs and the size of a dot implies the capacity of the corresponding FEN. The weight of an edge between two FENs is determined proportional to the physical distance between them. The corresponding logical fog network, shown in Figure 6b, was constructed based on the logical fog network construction mechanism described in Section 3.2.

For the performance comparisons of the proposed MC-SP and FC-SP mechanisms, we designed and implemented a mechanism, called the on-demand mechanism, that dynamically places the corresponding service image upon a request based on the logical fog network. In the on-demand mechanism, if an end device receives a request on a service, it checks whether it has any FENs installed with the corresponding service image within 2-hop in the logical fog network. If there is none, it checks whether any of its ancestor FENs have the service image. If none of the ancestor FENs have the service image, it places the corresponding service image on itself. In the case when it is the lack of the resources, it places the service image on the 2-hop FEN which is the closest to itself. If it is not possible, it places the service image on one of its ancestor FENs which is the closest to itself. By comparing our proposed mechanisms with the on-demand mechanism operating on the basis of per-service request, we measure the performance of ours in terms of the resource utilization of FENs.

**Figure 7:**
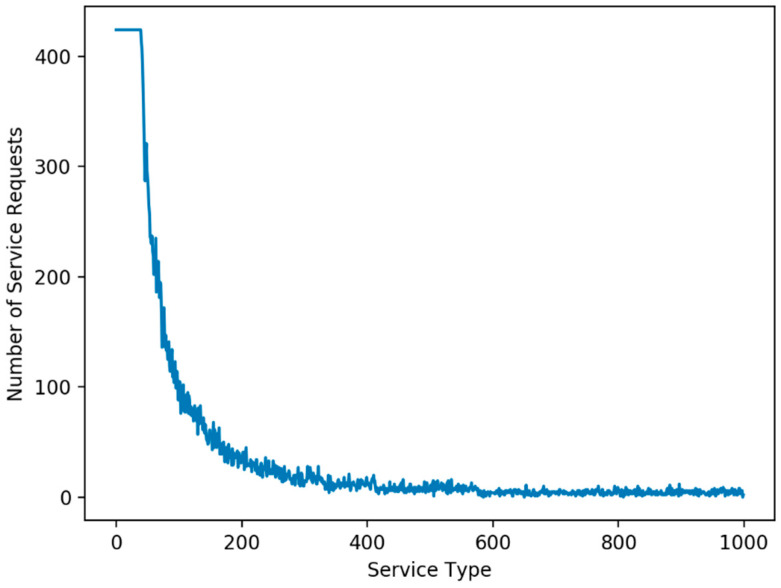
The L distribution case with 42,210 service demands for 1000 service types [12].

The number of service types, the distribution of service demands, the maximum capacity of CDC, and the maximum capacity of each FEN are the factors affecting performance. The number of service types requested by end devices is set to 1000 and 2000 service types and the number of end devices is 450. For the generation of service demands, we adopt the long-tailed distribution [24] of service demands in which a small set of service types is heavily requested and the rest of the service types are not frequently requested. The long-tailed distribution is adopted because a set of popular services are heavily used in the real world. For the simulations, the long-tailed distribution of service demands (we call this the L distribution case) is used, where about 10% of 1000 service types are heavily requested by the end devices, as shown in Figure 7 [12], which is obtained by using the zeta distribution [25]. This is achieved by using the function np.random.zipf (1.6, 1000) [26] where 1.6 is the value of the distribution parameter and 1000 is the number of service types. The function returns a value (we call this an L value) for each service type and the L value is used in determining the end devices with a service request for the service type. If the L value of a service type is greater than or equal to 100, all the end devices are assigned with a service request for the service type. Otherwise, the L value is used as the probability of assigning a service request for the service type to each end device. That is, for a service type, a higher L value implies a higher possibility of assigning a corresponding service request to each end device. If we sum up the service requests for all the service types in Figure 7, it becomes 42,210 in total.

The measured performance factors are the number of service images placed, the number of non-accommodated service requests, and the average network cost per service request. The average network cost per service request is the average one-way physical distance required for providing a service which is assumed to be proportional to the delay. For the calculation of the average network cost per service request, the physical distance between the end device with a service request and the FEN accommodating the corresponding service request is measured on the logical fog network. The physical distance is measured for each service request and then, all the measured physical distances are summed up. The average network cost per service request is obtained by dividing the total physical distance by the number of service requests. The maximum capacity of CDC is set to 300, 500 and 750 resource units, and one service image placement is assumed to require one unit of resources.

**Figure 8:**
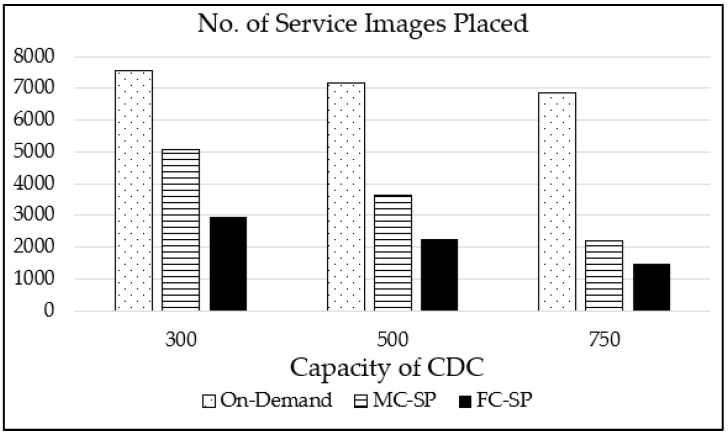
The number of service image placements for various maximum cloud data centers (CDCs) capacities with 1000 service types for the L distribution case.

**Figure 9:**
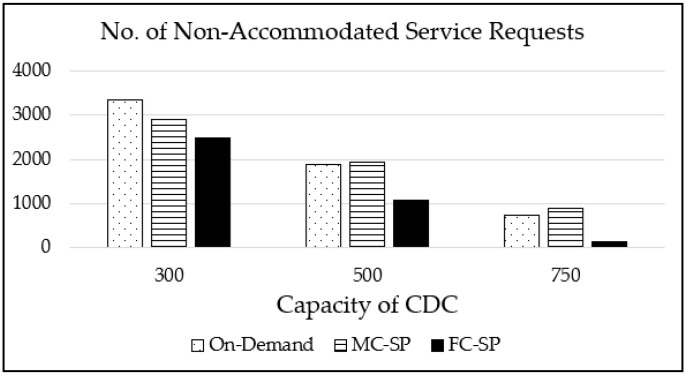
The number of non-accommodated service requests for various maximum CDC capacities with 1000 service types for the L distribution case.

**Figure 10:**
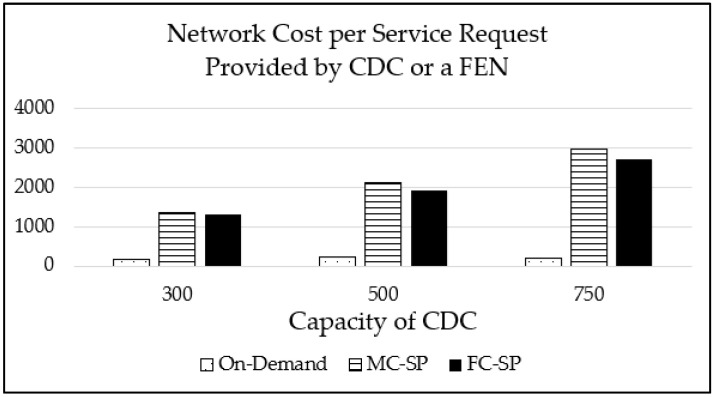
The network cost per service request for various maximum CDC capacities with 1000 service types for the L distribution case.

**Figure 11:**
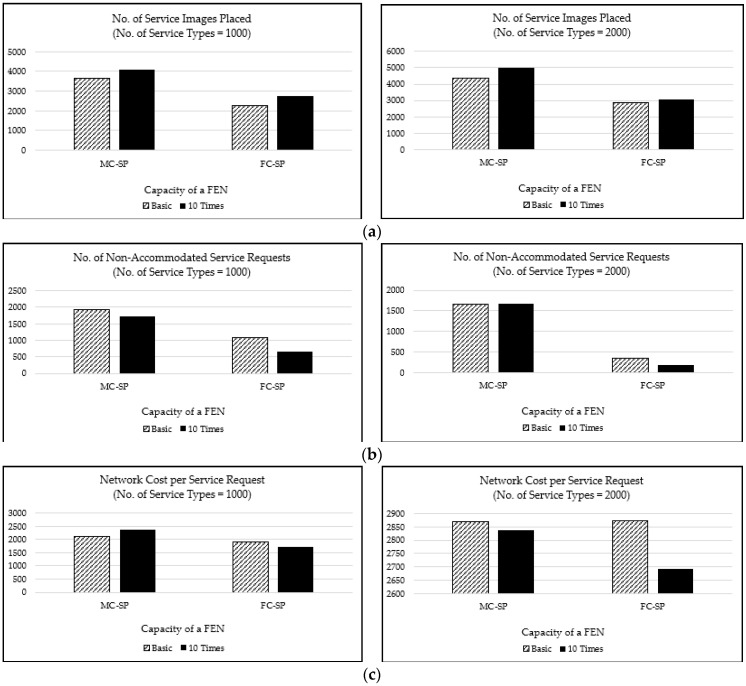
The performance according to the FEN capacity and the number of service types for the L distribution case: (**a**) the number of service images placed; (**b**) the number of non-accommodated service requests; and (**c**) the network cost per service request.

Figure 8, Figure 9, Figure 10 and Figure 11 show the performances of the proposed MC-SP and FC-SP mechanisms compared with the on-demand mechanism for various maximum CDC capacities of 300, 500 and 750 with 1000 services types for the L distribution case. Figure 8 depicts the graph showing the performance in terms of the number of service images placed on FENs. We can observe that, as the maximum capacity of CDC increases, more service images are placed on CDC, resulting in less service images placed on FENs in all the mechanisms. The on-demand mechanism places significantly more service images than our proposed mechanisms and even with increased CDC capacity, the on-demand mechanism slightly reduces the number of service images. The reason for this is that the on-demand mechanism places a service image near the end device which has requested the service, resulting in many service images near to end devices. On the other hand, the MC-SP and the FC-SP mechanisms reduce the number of service images placed significantly. Hence, we can assert that the FC-SP mechanism considering the service pattern performs the best in the resource utilization of FENs.

Figure 9 is the graph showing the number of service requests for which the corresponding service images are not placed on any FENs for the L distribution case. Thanks to the well-utilized FEN resources, the FC-SP significantly outperforms the MC-SP mechanism and the on-demand mechanism, especially for larger CDC capacities. As we expected, the number of non-accommodated service requests decreases as the maximum CDC capacity increases for all the mechanisms.

In Figure 10, the average network cost of handling a service request is shown for various maximum CDC capacities for the L distribution case. As the maximum CDC capacity increases, the network cost also increases because more service images are placed on the CDC. Both of the proposed mechanisms perform much worse than the on-demand mechanism because service images are placed near the end devices in the on-demand mechanism with less service images placed. As for the proposed mechanisms, the FC-SP mechanism requires less network cost than the MC-SP mechanism. The reason is that the MC-SP mechanism does not consider the pattern of service demands and places service images on FENs at higher levels of the logical fog network for larger coverage.

The performance of the proposed mechanisms is shown in Figure 11 with a varying FEN capacity and number of service types for the L distribution case. We simulated two cases of the FEN capacity, the basic case and the 10 times case. The basic case is the case of the FEN capacity in Figure 6b and the 10 times case is the FEN capacity of 10 times that of Figure 6b. The number of service types is set to 1000 and 2000.

Figure 11a shows the performance in terms of the number of service images placed on FENs, and Figure 11b shows the performance in terms of the number of non-accommodated service requests for the L distribution case. For the larger FEN capacity (i.e., the 10 times case), both MC-SP and FC-SP mechanisms place more service images, but the FC-SP mechanism decreases the number of non-accommodated service requests more significantly than the MC-SP. This indicates that the FC-SP mechanism fully utilizes the advantage of the increased FEN capacity. That is, the FC-SP mechanism accommodates more service requests than the MC-SP mechanism by provisioning more service images on FENs. In addition, as the number of service types increases, more service images are placed and more service requests are accommodated in both of the mechanisms. It is intuitive that more service images are placed for more service types, but the MC-SP mechanism shows a more noticeable increase in the number of service images placed with an insignificant decrease in the number of non-accommodated service requests. This implies that the MC-SP does not perform well in the situation of changed FEN capacities.

Figure 11c shows the graphs depicting the average network cost of a service request for the L distribution case. As the FEN capacity increases, the network cost decreases in both of the mechanisms. The FC-SP mechanism shows a more significant decrease in the network cost compared to the MC-SP mechanism, especially for more service types. This indicates that the FC-SP mechanism performs better than the MC-SP mechanism even for the case of more service types. Overall, the FC-SP mechanism is better than the MC-SP in utilizing the FEN resources and in adapting to changing environments such as the changes in CDC capacity, FEN capacity, and the number of service types to support.

**Figure 12:**
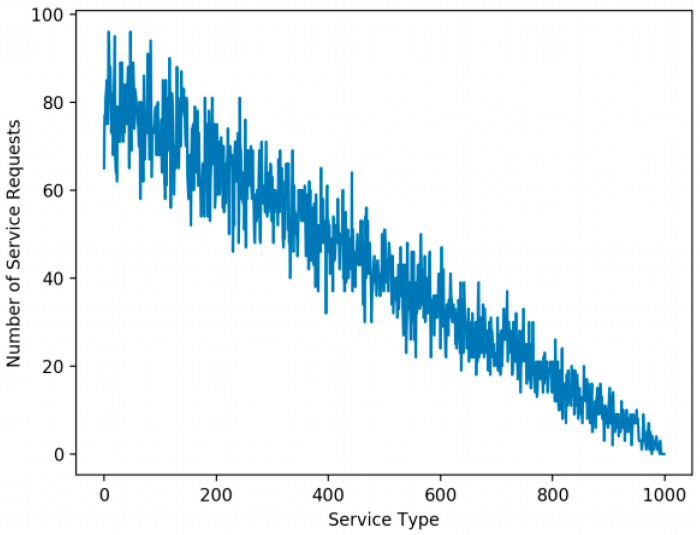
The U distribution case with 140,525 service demands for 1000 service types.

For a more concrete evaluation of the performance, we performed simulations for another service demand distribution shown in Figure 12 (we call this the U distribution case) which is obtained by using the uniform distribution. This is achieved by using the function np.random.uniform (0, 100, 1000) [27] where 0 and 100 are the lower and the upper boundary of the output interval, respectively, and 1000 is the number of service types. This function returns a value (we call this a U value) in the half-open output interval [0, 100) for each service type. The U value is used as the probability of assigning a service request for the service type to each end device. That is, the higher the U value of a service type is, the higher the possibility of assigning a service request of the service type to each end device. The U distribution case tends to assign service requests more evenly on service types than the L distribution case. The U distribution case generates 140,525 service requests for the performance evaluation of our mechanisms in a situation with heavy service requests.

**Figure 13:**
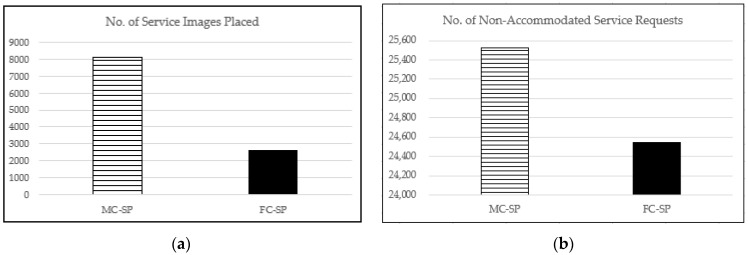

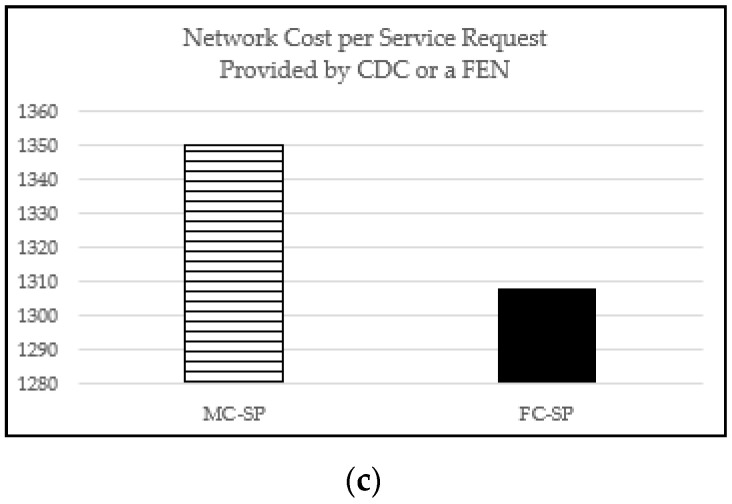
The performance with the maximum CDC capacity of 500 and 1000 service types for the U distribution case: (**a**) the number of service images placed; (**b**) the number of non-accommodated service requests; and (**c**) the network cost per service request provided by CDC or a FEN.

The performance of the proposed mechanisms for the U distribution case is depicted in Figure 13. We can easily see that the FC-SP mechanism outperforms the MC-SP mechanism in the aspect of all the performance factors. This indicates that the FC-SP mechanism performs better than the MC-SP mechanism even for the case with service requests relatively less biased over the service types (i.e., the U distribution case).

**Figure 14:**
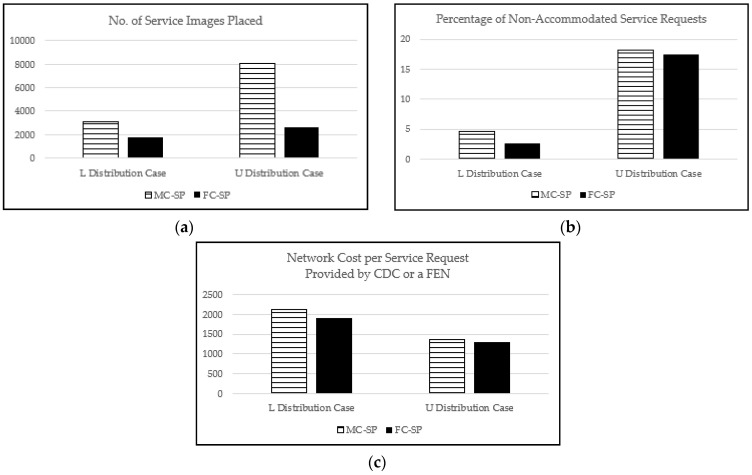
The performance comparision of the L distribution case and the U distribution case with the maximum CDC capacity of 500 and 1000 service types: (**a**) the number of service images placed; (**b**) the number of non-accommodated service requests; (**c**) the network cost per service request provided by CDC or a FEN.

From Figure 14, we can observe the performance comparison of our proposed SP mechanisms for the L and the U distribution cases. In Figure 14a,b, it is clearly shown that our mechanisms perform better for the L distribution case than for the U distribution case in terms of the number of service images placed and the percentage of non-accommodated service requests. This can be intuitively expected because the U distribution case has about 3.33 times more service requests than the L distribution case. In addition, we can see that the FC-SP mechanism performs very effectively in the sense that it accommodates more service requests than the MC-SP mechanism with many less service images placed, even in the stressful situation (i.e., the U distribution case). Figure 14c shows that the average network cost per service request for the U distribution case is lower than that for the L distribution case. The reason for this is that, for the L distribution case, the majority of the service requests are likely covered by higher-level FENs because most of the service requests are biased on a few specific service types, resulting in higher average network cost per service request than the U distribution case.

## 5. Conclusions

Because of the limited resources of FENs, it is not possible to place all the services on an FEN. Moreover, if service images are placed on FENs in a per service request-based way, the resources of FENs may not be efficiently utilized due to the duplicate placement of service images, resulting in less accommodation of service requests. Therefore, in this paper, we proposed two SP mechanisms, the MC-SP mechanism and the FC-SP mechanism, for provisioning service images on FENs on the basis of logical fog network considering the pre-obtained service demands. The MC-SP mechanism provisions service images on FENs based on the number of service requests from end devices and the FC-SP mechanism based on the locations of end devices requesting services. The performance of the proposed mechanisms was evaluated by carrying out through simulations. According to the simulation results, we observed that both of our mechanisms perform better than the On-Demand mechanism which was designed for performance comparison and operates in a simple manner of placing a service image near to the end device requesting the service upon each service request. Therefore, we can say that our mechanisms are scalable because they can save the FEN resources by effectively placing service images on FENs and are good for the environment with plenty of IoT devices deployed.

## Figures and Tables

**Table 1:** Notations for the definition of the optimal service provisioning (SP) problem.

Notation	Description
S	The set of services; $S=\;\left\{s_{1},\;\ldots ,\;s_{\alpha }\right\}$
F	The set of FENs; $F=\;\left\{f_{1},\;\ldots ,\;f_{\beta }\right\}$
U	The set of end devices; $U=\;\left\{u_{1},\;\ldots ,\;u_{\gamma }\right\}$
X	The service placement matrix; $,X=\;\left[x_{ij}\right]$
$x_{ij}$	The binary variable indicating whether a service image of the service $s_{i}$ is provisioned in the FEN $f_{j}$ or not; $x_{ij}=\;\left\{\begin{array}{c} 1,\;\mathrm{if}\;s_{i}\;\mathrm{is}\;\mathrm{provisioned}\;\mathrm{on}\;f_{j}\\ 0,\;\mathrm{otherwise} \end{array}\right.$
D	The given service demand matrix consisting of the service demands, $d_{ik}$’s
$d_{ik}$	The element of D indicating whether the service $s_{i}$ is demanded by the end device $u_{k}$ or not; $d_{ik}=\;\left\{\begin{array}{c} 1,\;\mathrm{if}\;s_{i}\;\mathrm{is}\;\mathrm{demanded}\;\mathrm{by}\;u_{k}\\ 0,\;\mathrm{otherwise} \end{array}\right.$
$c_{j}$	The maximum capacity of the FEN $f_{j}$
$T_{i}$	The maximum delay requirement of the service $s_{i}$
$\tau_{jk}$	The delay from the FEN $f_{j}$ to the end device $u_{k}$
$b_{ik}$	The binary variable indicating whether the delay requirement of the service demand $d_{ik}$ is satisfied or not; $b_{ik}=\;\left\{\begin{array}{c} 1,\;\mathrm{if}\;\mathrm{the}\;\mathrm{delay}\;\mathrm{requirement}\;\mathrm{of}\;d_{ik}\;\mathrm{is}\;\mathrm{satisfied}\\ 0,\;\mathrm{otherwise} \end{array}\right.$

**Table 2:** Notations for the SP mechanisms.

Notation	Description
$N_{fog}$	The logical fog network
$\overset{˘}{F}$	The ordered list of the FENs in F from the highest to the lowest level of $N_{fog}$; $\overset{˘}{F}=\langle f_{1}^{\prime },\ldots ,f_{\beta }^{\prime }\rangle$
$V_{fog}$	The coverage matrix of $N_{fog}$ consisting of $v_{kj}$’s
$v_{kj}$	The element of $V_{fog}$ indicating whether the end device $u_{k}$ is covered by the FEN $f_{j}^{\prime }$ or not; $v_{kj}=\left\{\begin{array}{c} 1,\;\mathrm{if}\;u_{k}\;\mathrm{is}\;\mathrm{covered}\;\mathrm{by}\;f_{j}^{\prime }\\ \;0,\;\mathrm{otherwise} \end{array}\right.$
W	The total demand matrix consisting of $w_{ij}$’s
$w_{ij}$	The element of W indicating the total demands on the service $s_{i}$ from the end devices covered by the FEN $f_{j}^{\prime }$
$W_{j}$	The jth column of W; $W_{j}=\langle W_{j}\left(1\right),\ldots ,W_{j}\left(\alpha \right)\rangle$, where $W_{j}\left(q\right)\;=w_{qj}$ for $q\in \;\left\{1,\ldots ,\alpha \right\}$
$\overset{˘}{W_{j}}$	The sorted list of $W_{j}$ in the decreasing order of the amount of demands; $\overset{˘}{W_{j}}=\langle \overset{˘}{W_{j}}\left(1\right),\ldots ,\overset{˘}{W_{j}}\left(\alpha \right)\rangle$, $\overset{˘}{W_{j}}\left(q\right)\geq \overset{˘}{W_{j}}\left(\overset{ˇ}{q}\right)$ if $q<\overset{ˇ}{q}$ for $q,\overset{ˇ}{q}\in \;\left\{1,\ldots ,\alpha \right\}$
$Y_{j}$	The corresponding service list of $W_{j}$; $Y_{j}=\langle Y_{j}\left(1\right),\ldots ,Y_{j}\left(\alpha \right)\rangle$, $Y_{j}\left(q\right)\in S$ for $q\in \left\{1,\ldots ,\alpha \right\}$

**Table 3:** Additional notations for the flexible coverage-SP (FC-SP) mechanism.

Notation	Description
Z	The ordered list of $z_{i}$’s; Z = <$z_{1}$, …, $z_{\alpha }$>, where $z_{i}\;$ is the total demands on $s_{i}$, $z_{i}=\underset{j\in \left\{1,\ldots ,\beta \right\}}{\sum }w_{ij}$
$\overset{˘}{Z}$	The sorted list of Z in the decreasing order of the total demands on services
$\overset{˘}{S}$	The corresponding service list of $\overset{˘}{Z}$; $\overset{˘}{S}=\overset{˘}{s_{1}},\ldots ,\overset{˘}{s_{\alpha }}$, where $\overset{˘}{s_{i}}$ is the service on which the total demands of $\overset{˘}{z_{i}}$ are imposed
$A^{srv}$	The ordered list of LCA FENs satisfying Condition-D of each service in $\overset{˘}{S}$
$A^{fog}$	The ordered list of the services with the same LCA FENs; the ith element, $A^{fog}\left(i\right)$, is the ordered list of the services, in the decreasing order of the total demands imposed on each service, whose LCA FEN is $f_{i}^{\prime }$
σ	The current service to be provisioned
$f^{min}$	The minimum entropy service which was already provisioned on the current FEN
$f^{p}$	The parent node of the current FEN
$f^{c}$	The child node of the current FEN
$P^{fog}$	The ordered list of visited ancestor FENs starting from $f_{i}^{\prime }$
